# Biomechanical assessment of unilateral/bilateral lumbar spondylolysis with and without muscle weakness using finite element analysis

**DOI:** 10.1016/j.heliyon.2025.e42647

**Published:** 2025-02-12

**Authors:** Baiyang Ding, Kazuhiro Imai, Zhuo Chen, Liang Xiao

**Affiliations:** a Department of Life Sciences, Graduate School of Arts and Sciences, The University of Tokyo, Komaba, Meguro-ku, Tokyo, Japan, 3-8-1, Komaba, Meguro-ku, Tokyo, 153-8902, Japan; b Spine Research Center of Wannan Medical College, Wuhu, China, 22, Wenchang West Road, China, Wuhu, 241001, China

## Abstract

**Background:**

Spondylolysis is commonly linked to low back pain in athletes, and the connection between muscle weakness and spondylolysis is unclear. Therefore, this study examined the biomechanics of spondylolysis and influence of muscle weakness by finite element (FE) analysis.

**Methods:**

A patient's L1–S1 lumbosacral unit was scanned by computed tomography, and generated a three-dimensional pathology-free FE model. Unilateral incomplete, unilateral, and bilateral defect models of the L5 isthmus were created. The mobility of the sacrum was limited to 7.5 N m moment, and a 500 N load was applied to the top of the L1 to simulate the human weight. The loading conditions included flexion, extension, bending, and axial torsion. Muscle forces representing the global back muscles and abdominal muscles, follower loads, and body weight were added to the FE model. The force of the global back muscles was decreased to 50 % to simulate back muscle weakness.

**Findings:**

The result shows that the L5 vertebral body in unilateral incomplete lumbar spondylolysis exhibited the greatest range of motion during flexion. The L5 vertebral body in unilateral lumbar spondylolysis demonstrated increased mobility during flexion and right torsion. The reduction of muscle strength led to the range of motion of L5 being reduced in all motions, but the maximum principal stress did not change significantly. However, the range of motion and maximum principal stress of L4 increased, especially in the bilateral group.

**Interpretations:**

From a biomechanical perspective, this study indicates that unilateral incomplete spondylolysis and unilateral spondylolysis might worsen during different motions. The reduction of muscle strength can cause spondylolysis to spread to adjacent segments and further worsen. A decrease in muscle force led to a decrease in the range of motion in the lumbar spondylolysis defect, but it also led to an increase in the range of motion in the adjacent segments.

## Highlights

• A finite element models of L1-S1 and muscle loading were constructed and validated.
• Unilateral incomplete spondylolysis may worsen to unilateral spondylolysis during axial rotation.
• Unilateral spondylolysis may worsen to bilateral spondylolysis during flexion.
• Back muscle weakness lead to, or aggravate range of motion and deterioration in adjacent segments.

## 1 Introduction

Lumbar spondylolysis is a bony defect of the pars interarticularis [1]. The most common type of lumbar spondylolysis is characterized by bilateral defects of the L5 vertebra [2,3]. Increased pressure on the pedicles can worsen the symptoms of lumbar spondylolysis and even lead to pedicle hypertrophy [4,5]. Spondylolysis defects are commonly considered fatigue fractures caused by repeated stress [6]. Repetitive stress on the lumbar spine caused by the repeated motions involved in sports might contribute to the occurrence of multi-level spondylolysis. Nonoperative and conservative therapy, such as flexion–extension strengthening exercises and stabilization exercises, has always been the first choice for lumbar spondylolysis [7,8]. Although surgical treatment is the final option, the effectiveness of conservative treatment in all types of lumbar spondylolysis remains unclear [9,10].

Studies have shown that the pars interarticularis is weak and has higher stress magnitudes than the other part of the vertebra, making it more susceptible to fracture [11,12]. Human cadavers and radiological techniques have limitations, such as their inability to simulate motion and many types of pathological changes [13]. Finite element (FE) studies have shown that stresses at the pedicle increase after vertebral fracture. Rami Haj-Ali et al. revealed models of unilateral and bilateral spondylolysis, but there are few models of special types of lumbar spondylolysis to prove the connection between reduction of muscle strength and lumbar spondylolysis, such as unilateral incomplete spondylolysis and muscle strength models [14]. In addition, there are muscles around the human lumbar spine. The presence of muscles affects the movement of the lumbar spine [15]. Combined with previous literature, lumbar spondylolysis causes spine instability and affects the muscles, decreasing overall muscle strength [16]. Previous literature has focused on the lumbar spine vertebrae and has not considered the influence of muscle strength [17]. Therefore, researching spondylolysis under muscle conditions can help these patients build greater rehabilitation plans. In the present study, we used computed tomography (CT) data from healthy volunteer and FE modeling to create a three-dimensional model of the lumbar. The FE model was used to study the differences and effects of segmental stability of different types of spondylolysis under conditions of muscle weakness. The objective of this study was to use FE analysis to compare the biomechanical characteristics of different types of lumbar spondylolysis and their influences on lumbar spondylolysis in the presence of muscle weakness. In this study, we analyzed the biomechanical characteristics of the incomplete lumbar spondylolysis model and investigated the effects of muscle weakness on different types of lumbar spondylolysis.

## 2 Methods

### 2.1 Patients and study design

**Supplementary figure 2:**
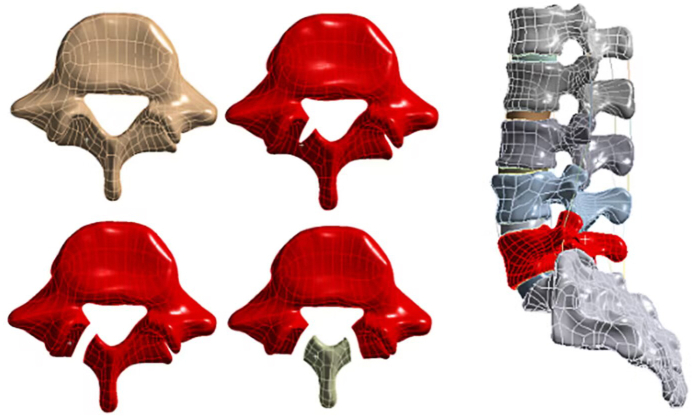
Construct 4 groups of CAD (geometric) models.

The protocol of this study was reviewed and approved in compliance with the declaration of the Helsinki statement (Notification Number 2023311). An FE model was constructed from the first lumbar vertebra to the first sacral vertebra. High-resolution lumbosacral spine CT data of a 26-year-old Chinese woman without spondylolysis were obtained from Wannan Hospital in Digital Imaging and Communications in Medicine file format. (weight 53 kg, height 161 cm) Written informed consent form has been obtained. First, the CT data were imported into image processing software (Mimics version 21.0; Materialise, Leuven, Belgium) to reconstruct the geometric model. Then, the L5 vertebral defect was created by deleting the module in the L5 partial area (2 mm) in SolidWorks 2017 (Dassault Systèmes, Versailles, France) [17]. Next, the models were divided into 4 groups as follows, the local and overall FE models are shown in Supplementary Figs. 1a, 1b, 1c and the CAD (geometric) models are shown in Supplementary Fig. 2.

Ⅰ. Complete L1–S1 spine model (Normal);
Ⅱ. L5 unilateral incomplete (isthmus) spondylolysis model (UNI incomplete);
Ⅲ. L5 unilateral (isthmus) spondylolysis model (UNI);
Ⅳ. L5 bilateral (isthmus) spondylolysis model (BIL);

The above models were analyzed and compared in range of motion (ROM) and the von Mises stress in ANSYS version 17.0 (Ansys Inc., Canonsburg, PA, USA). Finally, in order to study the effect of muscle loads on different spondylolysis models, the above 4 groups of models were added with muscle loads.

### 2.2 **Image segmentation and three-dimensional model generation from CT data**

**Supplementary Figure 3:**
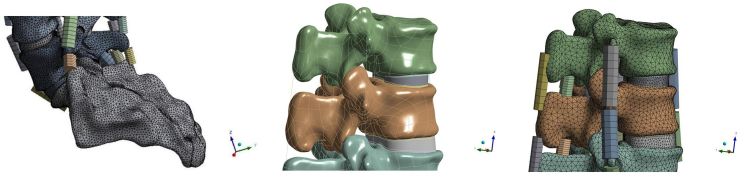
Construct the mesh of the model and the ligament.

The model file was imported into Geomagic Studio version 12.0 (3D Systems, Rock Hill, SC, USA) to perform model smoothing and eliminate excess triangular patches. The model file was imported into SolidWorks 2017 (Dassault Systèmes, Versailles, France) to solidify the model (node: 834,944, grid: 524,490). We uniformly scaled the model in Geomagic to create cancellous bone. The cortical bone thickness of the vertebrae was constructed to be 1.85 mm based on the measurements in the Mimics software [18,19]. The thickness of endplates on the superior and inferior surfaces of the intervertebral disc was constructed to be 0.5 mm [20]. In SolidWorks, the intervertebral disc is composed of the nucleus pulposus, annulus fibers, and cartilage endplate, with the nucleus pulposus accounting for 43 % of the total intervertebral disc [21,22]. Because each facet joint has a cartilage layer, we constructed a cartilage layer with a gap of 1.5 mm between the facet joints [23]. The L5 vertebral body defect was created by deleting pixels in the L5 partial region (2 mm). After smooth and accurate surface processing, the ligaments were meshed with hexahedral elements, and the other structures were meshed with tetrahedral elements using ANSYS version 17.0 (Ansys Inc., Canonsburg, PA, USA). Supplementary Fig. 3 shows that the mesh of model and the ligament. Unilateral vertebral defects were represented by left-sided defects (node: 620,220, grid: 381,506). Unilateral incomplete vertebral defects were assumed to be 50 % of complete defects (node: 833,793, grid: 523,774). Bilateral vertebral body defects were located at the left and right facet joints (node: 832,602, grid: 522,901). The ligaments of the lumbar spine were constructed according to their anatomical position in the human body. The type element of the ligament is the link, which only bears tension. These elements of the ligament do not bear any compression. The ligament properties are set from the studies of Chen, C.S and Cheng-Cheng Yu [24,25]. The cross-sectional area was then added in ANSYS to complete the construction and simulation of the ligament. To reduce the deviation between different spondylolysis groups, we modeled them as homogenous. Supplementary Table 1 shows the material properties of different tissues in the FE model.

### 2.3 Mesh convergence test and material properties sensitivity analysis

Mesh convergence test and material property sensitivity analysis are important for linear models [26]. The mesh convergence test was performed on one of the two FE models (without muscle load). Six mesh resolutions were generated consecutively for the FE model (in the order of unit size was automatic system division, 5, 4, 3, 2.5, and 2 mm). The von Mises stress of different tissues in the FE model was calculated and compared. When the predicted results of the two mesh schemes differences less than 5 %, the mesh was considered to be converged [27]. For sensitivity analysis of material properties, a healthy lumbar spine model in this study was tested in flexion (7.5 N m) direction. The parameters were linearized according to the previous studies [28]. The studies by Jebaseelan et al. (2012) showed that the material properties of the annulus fibers are not sensitive and the material properties of the ligaments are highly nonlinear [29]. Therefore, the sensitivity of the annulus fibers and ligaments was not tested in this study. In the sensitivity analysis, the models were divided into the linearized basic model, nonlinear model, low-value model (reduced by 25 % on the original parameters), and high-value model (increased by 25 % on the original parameters). In this study, the ROM, intradiscal pressure (IDP) and facet joint force of L1-L2 obtained by the linearized basic model, nonlinear model, and high- and low-value model were compared.

### 2.4 Boundary and loading conditions

First, the model was simulated in a standing position for reference. The sacroiliac joint of the sacrum was fixed to verify the movement and flexibility of the model. Based on the methods in the previous study and to maintain consistent with the subsequent analysis, a load of 500 N and a moment of 7.5 N m were applied to the top of L1 to simulate motion with the direction along the spine axis. Next, four static loading represent different activities (flexion, extension, bending, and axial rotation) were simulated [14,30]. The sagittal ROM, transverse axial ROM, the von Mises stress of the L4–L5 segment and the maximum principal stress were analyzed. The experimental data were compared with the literature and in vitro data to determine the rationality of the model. Finally, model data for healthy, unilateral incomplete, unilateral complete, and bilateral L5 lumbar defects were compared [31,32].

### 2.5 Muscle loading conditions

The muscle loads in this study adopted loading conditions implemented by Zhu et al. [33]. Four groups of muscle loading were applied to the FE model. All muscle loads were applied to the global spine model starting from L1. A 170-N force representing the global back muscle load was applied to the erector spinae 40 mm dorsal to the center of the disc. A 20-N force representing the global abdominal muscle load was applied to the rectus abdominis 153 mm ventral to the center of the disc. To maintain the same weight strength with the unloaded muscle, a weight of 500 N was added above 30 mm ventral to the upper disc. To represent the compressive effect of local muscle forces, a follower load of 200 N was added to the model. To simulate weakening of the back muscles, the force of 170 N was reduced by 50 % to 85 N. Previous studies that considered actual spinal geometry applied follower load to simulate muscle forces [34]. The ROM of L4-L5 was calculated to compare different types of lumbar spondylolysis with the sacrum fixed. All simulations were performed using the FE program ANSYS. The FE model of the lumbar region after the addition of further muscle loads is shown in Supplementary Fig. 4a. Supplementary Fig. 4b shows both sides of the sacroiliac joint fixed and the force added.

## 3 Results

### 3.1 FE model validation

**Supplementary figure 5:**
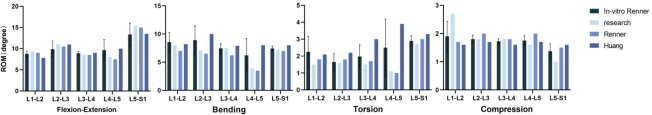
Comparison of the ROM of each motion segment between the current and previous studies.

**Supplementary figure 6:**
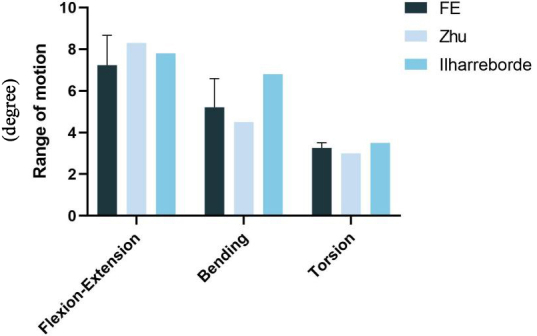
Comparison of the ROM of L5 segment between the current and previous studies under reduced muscle strength.

**Supplementary figure 7:**
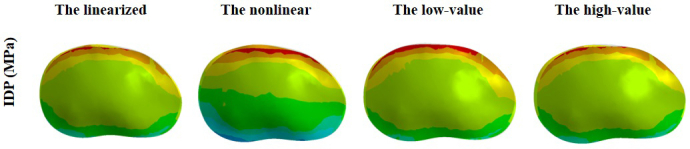
Material properties sensitivity analysis in intradiscal pressure.

The rationality of the FE model was verified according to the results of a study by Renner et al. and the experimental methods described by Huang et al. [31,32] Pure moments (7.5 N m) were applied to the center of the upper surface of the L1 vertebra in flexion, extension, bending, compression, and torsion, and each segment was compared with the results of the studies in vitro. As shown in Supplementary Fig. 5, our results matched the data from prior studies. Therefore, we believe that the FE model of this study is suitable for subsequent studies and those incorporating additional muscle. In addition, we also verified the rationality of the FE model after muscle loading according to the research results of Zhu et al. and the loading conditions described by Ilharreborde et al. The results are shown in the Supplementary Fig. 6. In the Mesh convergence test, The number of elements and nodes for each mesh resolution are shown in Supplementary Table 2. The six mesh resolutions in this study were tested by applying 50 N to the L1 surface after fixing S1. When the unit size was 3 mm, the stress result was 31.444 MPa; when the unit size was 2.5 mm, the stress result was 30.925 MPa. The difference between the two stress results was calculated to be 1.65 %. The results conform to the requirements of the mesh convergence test. In material property sensitivity analysis, the linearized parameters of the material properties in the model are shown in Supplementary Table 3 after parameter linearization. Supplementary Fig. 7 shows material properties sensitivity analysis in IDP.

ROM: The difference between the linearized basic model and the nonlinear model is 2.15 %;

The linearized basic model increased by 16.4 % compared with the low-value model;

The linearized basic model decreased by 14.4 % compared with the high-value model

IDP: The difference between the linearized basic model and the nonlinear model is 1.63 %;

The linearized basic model increased by 9.4 % compared with the low-value model;

The linearized basic model decreased by 10.3 % compared with the high-value model.

Facet joint force: The difference between the linearized basic model and the nonlinear model is 0.03 %;

The linearized basic model increased by 5.4 % compared with the low-value model;

The linearized basic model decreased by 20 % compared with the high-value model.

From the results, the verification in ROM and IDP is close to that in the previous studies.

### 3.2 Comparison of various lumbar spondylolysis models

#### 3.2.1 ROM

**Fig. 1:**
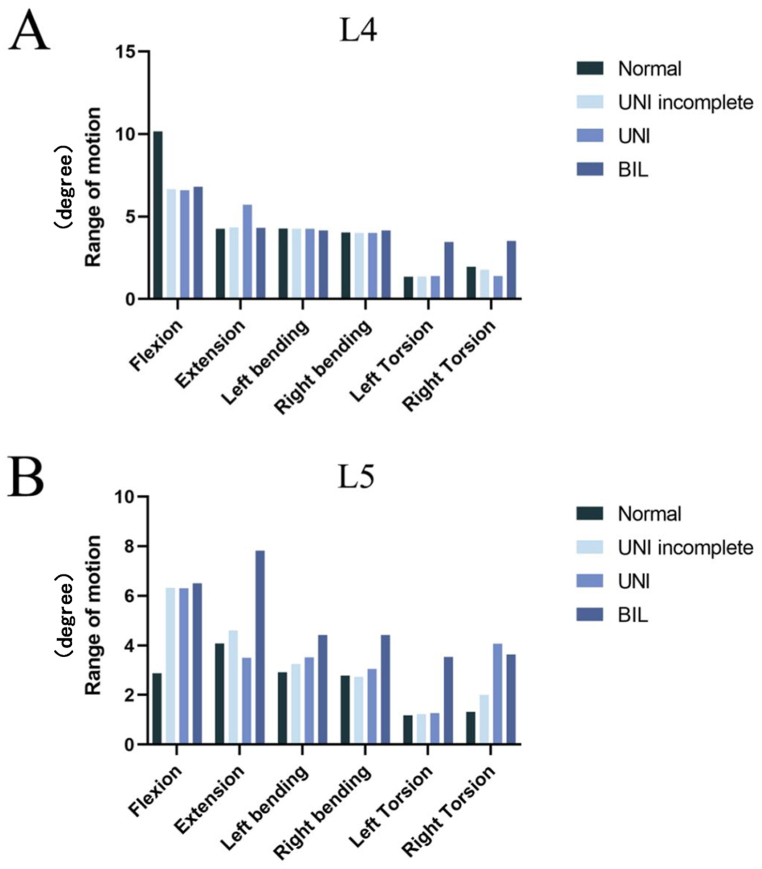
ROM of the L4 and L5 segments in flexion, extension, left/right bending, and left/right torsion in different lumbar spondylolysis models.

Fig. 1 shows the ROM of L4 and L5 lumbar segments in flexion, extension, left/right bending, and left/right torsion in different lumbar spondylolysis models. The detailed data are in Supplementary Table 4. The summary is as follows.

• Flexion: Compared with the normal group, the ROM of L5 in the defect group (unilateral incomplete lumbar spondylolysis and unilateral lumbar spondylolysis) increased by 118 %, and the ROM of bilateral lumbar isthmic spondylolysis was the largest (125 %). However, compared with the normal group, the ROM of L4 in the defect group decreased by about 35 %.
• Extension: L5 in the unilateral lumbar spondylolysis model decreased by about 14 % compared with the normal group. The ROM was largest in the bilateral lumbar spondylolysis group, increasing by about 90 %.
• Bending: Compared with the normal group, L5 in the bilateral lumbar spondylolysis model increased significantly by about 50 % during left and right lateral bending motions. In the unilateral lumbar spondylolysis model, the ROM of L5 during left and right bending motions was 20 % higher than that in the normal group. Interestingly, the ROM for unilateral incomplete lumbar spondylolysis coincided with that for unilateral complete lumbar spondylolysis during flexion and extension motions.
• Torsion: Because the left side was the defect side, there were no significant changes with left torsion motions in models except for the bilateral group. The ROM of L4 and L5 in the bilateral defect model increased by 156 % and 200% in the left torsion. However, different trends were found between the unilateral incomplete group, unilateral group, and bilateral group. The ROM of L5 in the unilateral incomplete group increased the least (40 %).

#### 3.2.2 The von Mises stress and the maximum principal stress

**Fig. 2:**
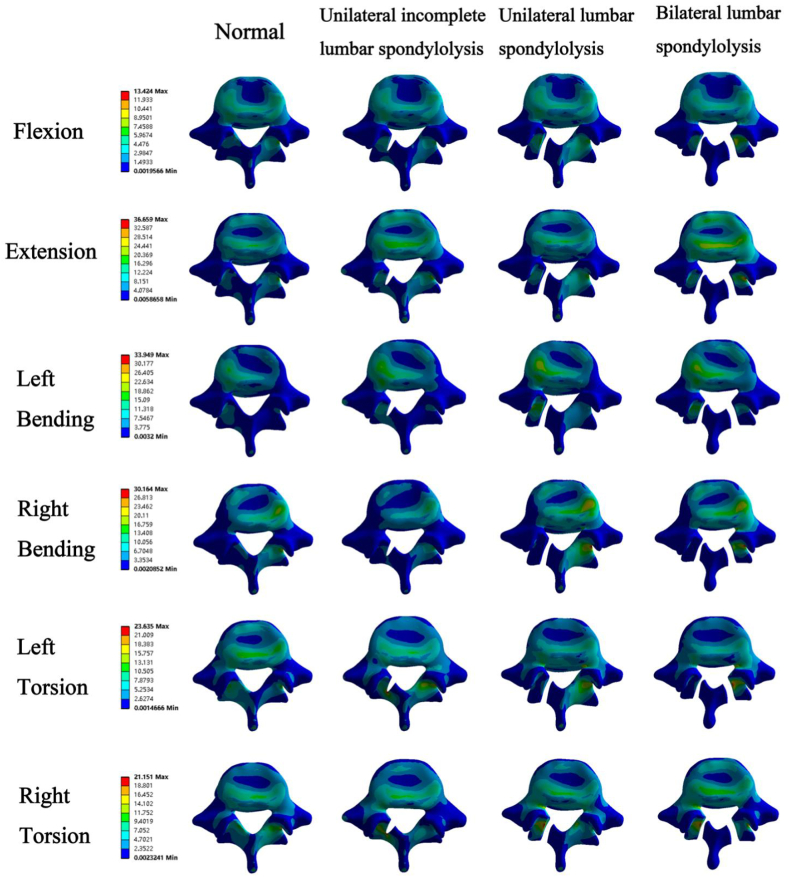
The von Mises stress of the L5 segment in different lumbar spondylolysis models.

**Fig. 3:**
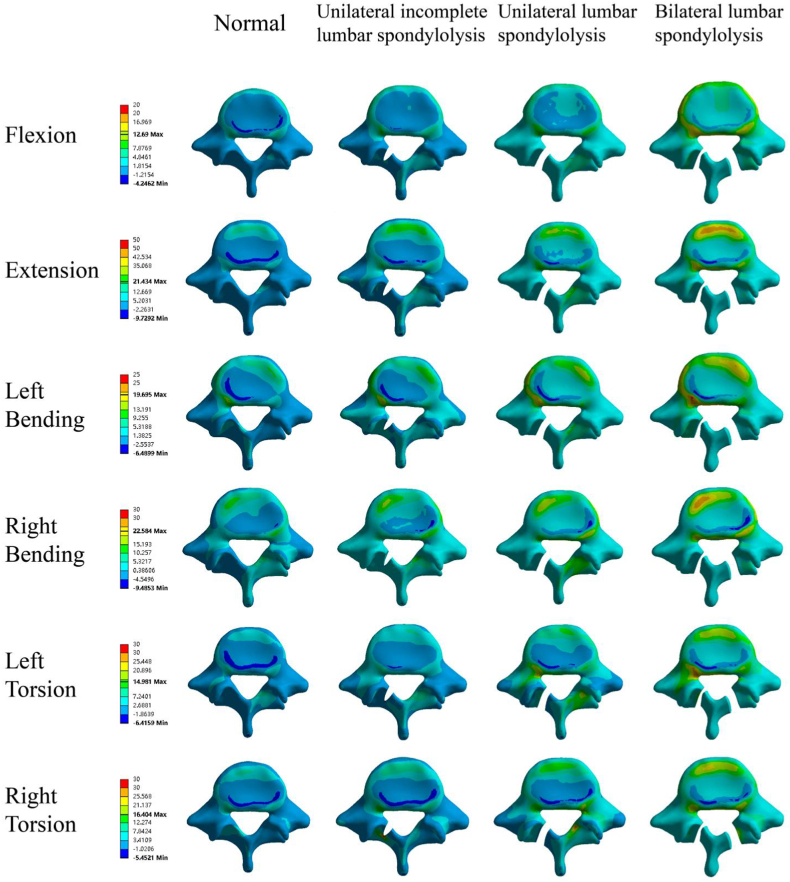
The maximum principal stress of the L5 segment in different lumbar spondylolysis models.

The von Mises stress of L5 caused by different motions in the lumbar FE model was compared with that of L5 in each defect model (Fig. 2). Consistent with the ROM results, The von Mises stress in the bilateral group changed significantly in all motions. The von Mises stress at the facet joint on the right side during left bending, and right bending in the unilateral models increased by about 80 % and 130 %. Interestingly, however, the unilateral incomplete group and unilateral group showed different trends in the von Mises stress during torsion. Among the various motions, the overall pressure increased by 50 % in flexion; the pressure on the left side increased by 50 % in extension, left bending, and torsion compared to the normal group; and the pressure on the right side in the unilateral group increased by 40 % in right bending. The results of the maximum principal stress (Fig. 3) showed that compared with the normal group, the maximum principal stress in the unilateral incomplete group was concentrated at the left fracture site during left flexion. The maximum principal stress in the unilateral group increased significantly at the right facet joint in the right torsion.

#### 3.2.3 Intradiscal pressure (IDP)

**Fig. 4:**
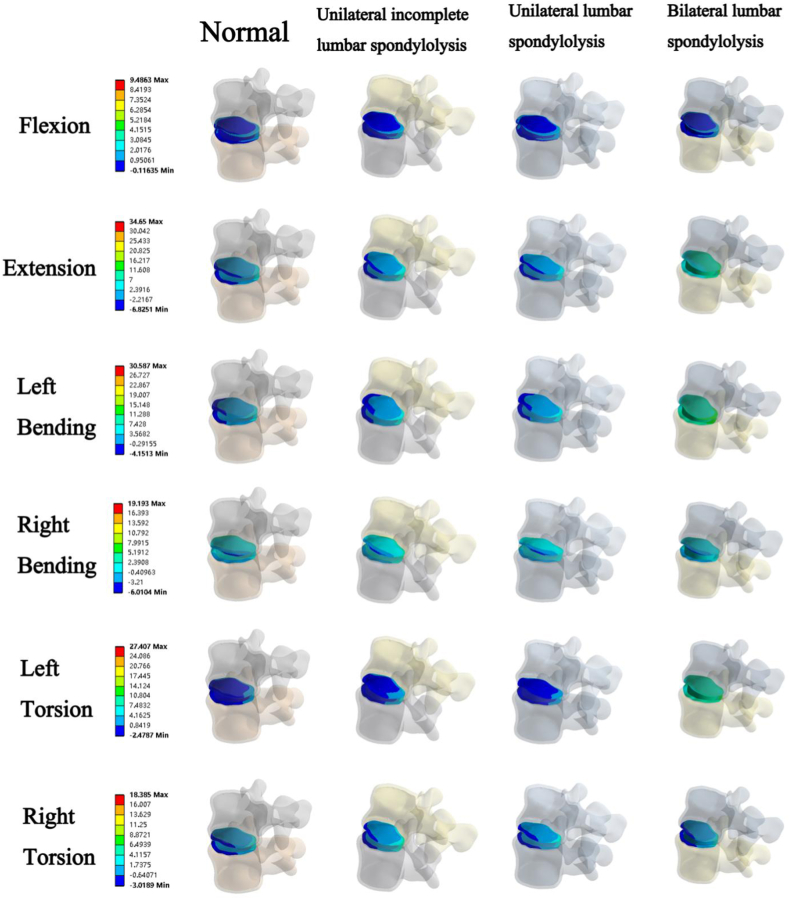
The L4/5 Intradiscal pressure in different lumbar spondylolysis models.

The changing trends and stress distribution of the L5/S IDP under different models are shown in Fig. 4. Except in flexion, right bending, and torsion, the bilateral group had a larger IDP than the normal group. During extension, left bending, and torsion, the IDP increased by 57 %, 60 % and 42 % in the bilateral group.

### 3.3 ROM of FE model under reduced muscle forces

**Fig. 5:**
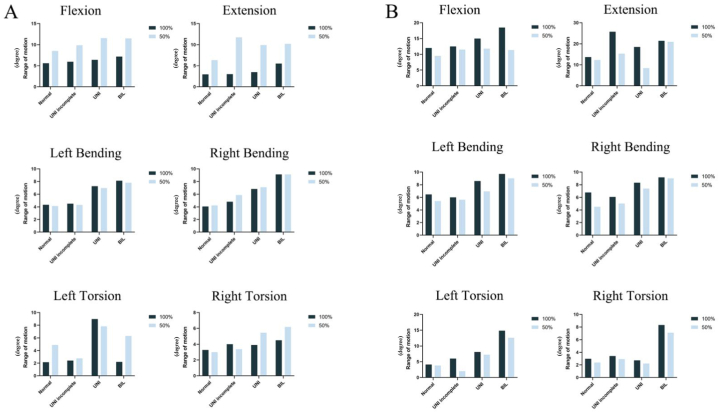
ROM of the L4 and L5 segments under normal and decreased muscle strength.

As shown in Fig. 5, the ROM of the defect (L5) was decreased in all motions because of the reduction of muscle forces in the normal, unilateral incomplete, unilateral, and bilateral groups. The detailed data are in Supplementary Table 5. All compared models were under conditions of muscle loading. Specific results are summarized as follows (Fig. 5A shows the data of L4, and Fig. 5B shows the data of L5).

• Flexion: When the muscle forces were reduced by 50 %, the ROM of L5 in the normal, unilateral incomplete, unilateral, and bilateral groups decreased by 20 %, 8 %, 21 %, and 38 %, respectively, and the ROM of L4 increased by 51 %, 63 %, 80 %, and 60 %, respectively.
• Extension: When the muscle forces were reduced by 50 %, the ROM of L5 in the normal and bilateral groups did not change significantly, but that of L4 increased by 115 % and 85 %. Interestingly, when the muscle forces were reduced by 50 %, the ROM of L5 in the unilateral incomplete and unilateral groups decreased by 40 % and 55 %, respectively, and that of L4 increased by 291 % and 185 %, respectively.
• Bending: When the muscle forces were reduced by 50 %, the ROM of L5 was reduced in all models except the bilateral group. During left bending, the ROM of L5 in the unilateral group decreased by 19 % because of the reduction in muscle forces. Interestingly, during right bending, the normal group was more strongly influenced by 34 %.
• Torsion: When the muscle forces were reduced by 50 %, the ROM of L4 in the normal and bilateral groups increased by 126 % and 186 %, respectively, during left torsion. However, the ROM of L4 in the unilateral incomplete group did not change significantly, while the ROM of L4 in the unilateral group decreased by 13 %. Unexpectedly, although the ROM of L5 decreased in all models, the decrease in the unilateral incomplete group was the most significant by 66 %. During right torsion, the ROM of L4 increased by 40 % and 37 % in the unilateral and bilateral groups.

#### 3.3.1 The maximum principal stress

**Fig. 6:**
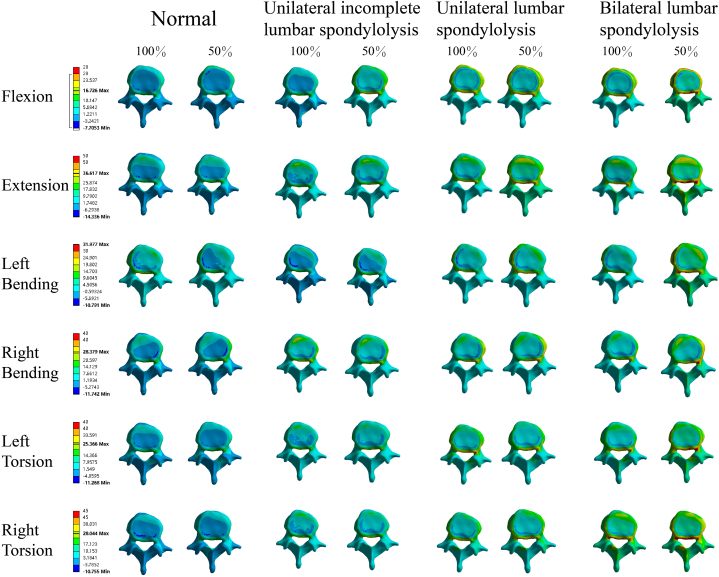
The maximum principal stress of the L4 segment under normal and decreased muscle strength.

The maximum principal stress (Fig. 6) showed that when the muscle load was reduced by 50 %, compared with the normal muscle load group, the maximum principal stress at the facet joints of the unilateral incomplete group and the unilateral group increased by 35 % and 39 % in L4 in the flexion, respectively. In the torsion motions, the maximum principal stress at the facet joints of the bilateral group increased by 60 %.

## 4 Discussion

Spondylolysis is generally considered a stress fracture phenomenon that is often related to disc degeneration or muscle weakness [35,36]. Osterman et al. found that 90 % of patients with lumbar spondylolysis showed involvement of L5, and Sakai found that all four patients with multi-level spondylolysis in their study had L5 spondylolysis [37,38]. Zhu investigated the methodical parameters of muscle force in an FE model [39]. Zhu et al. showed that more parameters should be considered in biomechanical models and that reducing the force of the global back muscles might lead to spondylolysis [33]. The question of whether muscle weakness is a cause or consequence of lumbar spondylolysis remains unresolved because of the complex connection between muscles and the lumbar spine [40]. Although there are many different types of spondylolysis, few studies have focused on incomplete lumbar spondylolysis [41]. In addition, Spondylolysis rehabilitation focuses on improving muscle stability, reactivating muscles, and resolving imbalances to treat muscle strength and function. And decreased muscle strength will affect the spondylolysis treatment. Therefore, researching spondylolysis under the decreased muscle strength condition might help these patients build greater rehabilitation plans [42]. This emphasizes the importance of using a conservative treatment to treat spondylolysis to prevent and reduce muscle decrease. Therefore, the research on muscle strength has clinical relevance.

The erector spinae as the main muscle of the lumbar spine, supports the whole back and even includes most of the spine. These back extensor muscles might also change due to the lumbar spine movement. Because the erector spinae are closely related to the lumbar spine movement, other muscle strength have an influence but is very small compared to the erector spinae. We only reduced the whole back muscle strength to reduce the influence of other muscle strength [43]. Although this is an unreal simulation, our purpose is to analyze the trend of spondylolysis biomechanics when muscle strength is reduced. Therefore, to be consistent with Rohlmann and Zhu's research, we reduced the strength of the back muscles to simulate the reduction of muscle strength [44]. At present, Zhu et al. provided us with muscle load conditions for research. Decreased muscle strength is inevitable with time and exercise. Insufficient rehabilitation will increase the reduction of muscle strength. Long-term rest for treating spondylolysis will lead to decreased muscle strength. Therefore, the main objective of this study was to reveal the biomechanics of the incomplete spondylolysis model, and the reduction of muscle strength can cause spondylolysis to spread to adjacent segments and further worsen.

The lumbar spondylolysis model without muscle showed that most differences occurred at the L5–S1 level, where the vertebral body defect was located. During flexion, the spondylolysis model showed a reduced ROM at L4–L5. We consider that this occurred because the L5 defect in flexion affects the mobility of L4. The right vertebra sustained greater stress when right axial torsion was applied to the completely fractured vertebra on the left side because the pars of the complete fracture lacked tensile resistance. This is also related to the reduced stability caused by the defect [45]. Mircea Sopon et al. also showed that axial strain mainly occurred after the fracture was produced [46]. However, the bilateral lumbar spondylolysis model showed an overall significant increase in ROM compared with the other models. The von Mises stress of the vertebral body, the maximum principal stress and nucleus pulposus and the IDP were also compared for each load case. In the incomplete unilateral lumbar spondylolysis, the right pars interarticularis of the L5 vertebra underwent greater stress during left and right axial torsion, but there were no significant changes during other motions. We consider that this occurred because the incomplete fracture absorbed most of the load. This might also be explained by the increase in stress at incomplete fracture locations during left and right axial torsion. The IDP in the study by Haj-Ali was consistent with that in our study, and lumbar spondylolysis with multiple fractures led to higher stress concentration in the disc except during flexion.

The results of the model remained consistent after the addition of muscles. This study showed that L5 lumbar spondylolysis under muscle weakness more strongly affects L4 than the motion segment of a healthy lumbar spine. In other FE studies, multiple fractures of the pars interarticularis affected the stability of adjacent motion segments [14]. During flexion motions in the present study, all lumbar spondylolysis models showed an increase in the ROM of L4 and a decrease in the ROM of L5. This is also explained by the reduction in muscle strength. Satoshi Kato et al. also concluded that vertebral fractures were associated with lower muscle strength [47]. The decrease in muscle strength leads to a decrease in the ROM of the defect. Regardless of the type of lumbar spondylolysis, muscle reduction will influence the adjacent lumbar segment. During bending motions, the ROM of L5 still decreased, but the ROM of L4 did not. This might have occurred because the muscle support on the left and right sides changes the force of L4. During torsion, the ROM of L4 increased in the normal and bilateral spondylolysis groups. Although the ROM of L5 decreased, the reduction in the unilateral incomplete group was the most significant. We believe that this might have been caused by the instability of the unilateral incomplete defect. Linus Lee et al. showed that pelvic fractures can cause pain and functional impairment due to the mechanical instability of the pelvic ring [48]. This also confirms the defects due to instability. Because of the changes in the ROM of L4, we conducted a maximum principal stress on L4. The results of the maximum principal stress of L4 were also consistent with the trend of ROM. We consider that multiple spondylolysis in L5 might affect the facet joints of adjacent segments, and this is related to muscle strength. Hong Jin Kim et al. demonstrated that the muscle volume around multiple fractures was lower than that around single fractures. Therefore, bilateral spondylolysis might lead to a decrease in muscle strength [49]. And a decrease in muscle strength will increase the possibility of adjacent segments developing into spondylolysis.

Like previous studies, this study has several limitations. Loading conditions and their combinations cannot include all possible loading conditions experienced during various motions in sports and daily life. Another limitation is that the model was idealized. The strength of the muscles was varied in a parametric way using data from previous studies. Thus, the results in the present study should be viewed as a comparative analysis. The correlation between back muscle weakness and spondylolysis needs more clinical research and biomechanical analysis. Although these forces do not need to use too much in vivo or in vitro data, comparing the contact forces under the same assumptions can provide some relative trends at least to some degree. However, in this study, the forces and moments provided by the boundary conditions were a simplified ideal situation. M. Dreischarf's study used different moments to test, while Song's study used the same moment to verify the model's validity [50]. To keep consistent with the content of subsequent analysis, we used the method of Song, Rami Haj-Ali et al. [14,30] Also, a follower load of 200 N was applied along the load path to minimize the influence of shear force. In addition, it is important to assess changes in load due to spondylolysis, but additional information on the connection between exercise and motions might be required. For example, sports such as golf and baseball, which might direct larger mechanical forces to the lumbar region, need to be studied by dividing the sports into different phases [51,52].

## 5 Conclusions

From a biomechanical perspective, unilateral incomplete spondylolysis may worsen to unilateral spondylolysis during axial rotation. Unilateral spondylolysis might worsen to bilateral spondylolysis during flexion. The reduction in muscle strength is the cause of spondylolysis to worsen. The reduction in muscle force led to a decrease in the ROM in the lumbar spondylolysis defect, but it also led to an increase in the ROM in the adjacent segments. This increases the risk of spondylolysis in adjacent segments.

## CRediT authorship contribution statement

**Baiyang Ding:** Writing – original draft, Methodology, Investigation. **Kazuhiro Imai:** Writing – review & editing, Supervision, Project administration. **Zhuo Chen:** Investigation, Data curation. **Liang Xiao:** Data curation.

## Declaration of competing interest

The authors declare that there are no conflict of interests, we do not have any possible conflicts of interest.

## Figures and Tables

**Supplementary figure 1a:**
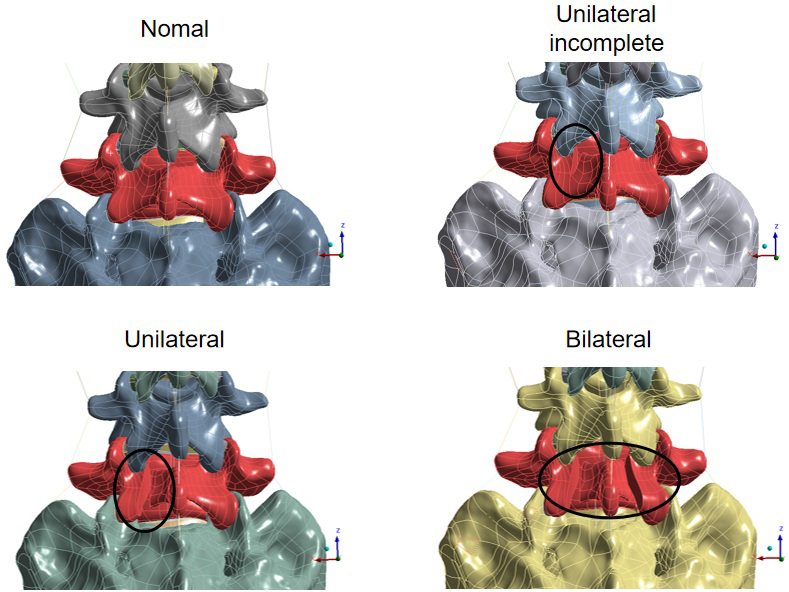
Construct 4 groups of local FE models.

**Supplementary figure 1b:**
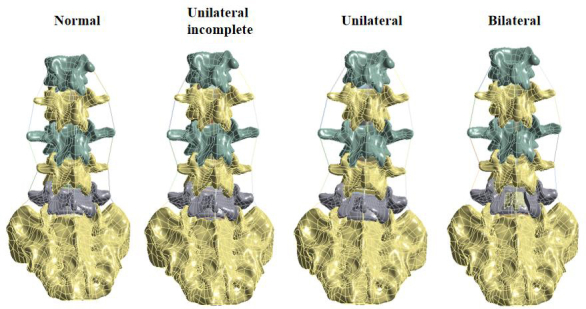
Construct 4 groups of global FE models.

**Supplementary figure 1c:**
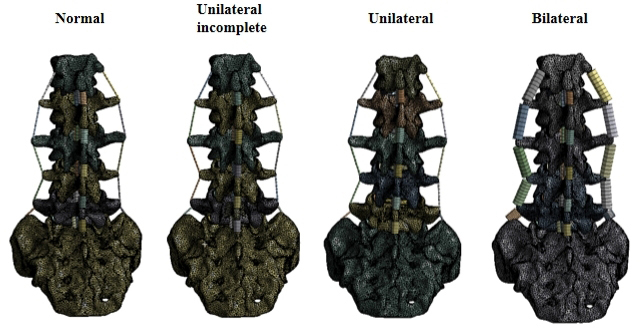
Construct 4 groups of global FE models with mesh units.

**Supplementary Figure 4a:**
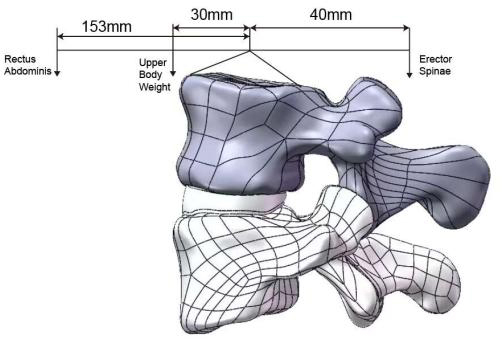
FE model of the lumbar with the applied muscle loads.

**Supplementary Figure 4b:**
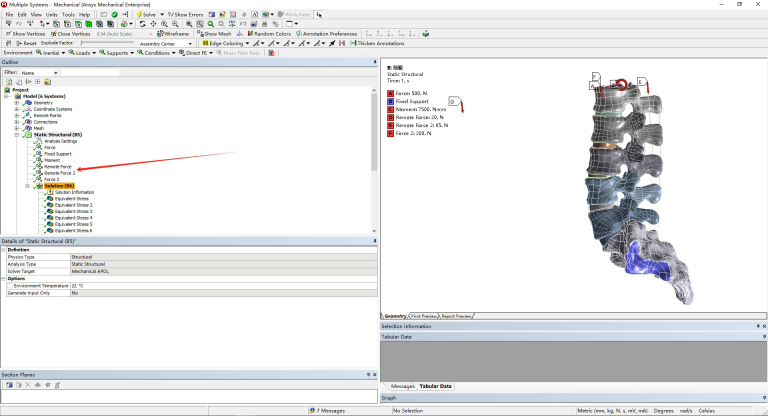
Fix both sides of the sacroiliac joint and add forces.
