# Supplementary material for: Biomechanical assessment of unilateral/bilateral lumbar spondylolysis with and without muscle weakness using finite element analysis
Source: Heliyon. 2025 Feb 12;11(4):e42647. doi: 10.1016/j.heliyon.2025.e42647 (PMC11891713; doi:10.1016/j.heliyon.2025.e42647)
Supplement: Multimedia component 1 [file mmc1.docx]

**Supplementary table. 1** Material properties used by finite element model.

| **Components** | **Young’s modulus (MPa)** | **Poisson’s ratio** | **Element type** | **Crosssectional area (mm2）** | **References** |
| --- | --- | --- | --- | --- | --- |
| **Vertebra** |  |  |  |  |  |
| Cortical bone | 12000 | 0.3 | Tetra |  | [Eric Wagnac](https://pubmed.ncbi.nlm.nih.gov/?term=Wagnac+E&cauthor_id=22566121) et al., 2012 |
| Cancellous bone | 100 | 0.2 | Tetra |  | [Eric Wagnac](https://pubmed.ncbi.nlm.nih.gov/?term=Wagnac+E&cauthor_id=22566121) et al., 2012 |
| Endplate | 24 | 0.4 | Tetra |  | Rohlmann A et al.,2006b |
| Sacrum | 5000 | 0.2 | Tetra |  | Huang et al.,2016 |
| Facet cartilage | 11 | 0.2 | Tetra |  | [V K Goel](https://pubmed.ncbi.nlm.nih.gov/?term=Goel+VK&cauthor_id=3061028) et al.,1988 |
| Annulus | 110 | 0.3 | Tetra |  | Rohlmann A et al.,2006b |
| Nucleus pulposus | 1 | 0.49 | Tetra |  | Rohlmann A et al.,2006b |
| **Ligament** |  |  |  |  |  |
| ALL | 10 | 0.3 | Hex | 63.7 | [Cheng-Cheng Yu](https://pubmed.ncbi.nlm.nih.gov/?term=Yu+CC&cauthor_id=27345748) et al.,2016 |
| PLL | 10 | 0.3 | Hex | 20 | [Cheng-Cheng Yu](https://pubmed.ncbi.nlm.nih.gov/?term=Yu+CC&cauthor_id=27345748) et al.,2016 |
| LF | 1.5 | 0.3 | Hex | 40 | [Cheng-Cheng Yu](https://pubmed.ncbi.nlm.nih.gov/?term=Yu+CC&cauthor_id=27345748) et al.,2016 |
| CL | 10 | 0.4 | Hex | 30 | [Cheng-Cheng Yu](https://pubmed.ncbi.nlm.nih.gov/?term=Yu+CC&cauthor_id=27345748) et al.,2016 |
| ISL | 1.5 | 0.3 | Hex | 40 | [Cheng-Cheng Yu](https://pubmed.ncbi.nlm.nih.gov/?term=Yu+CC&cauthor_id=27345748) et al.,2016 |
| SSL | 10 | 0.3 | Hex | 30 | [C S Chen](https://pubmed.ncbi.nlm.nih.gov/?term=Chen+CS&cauthor_id=11574255) et al.,2001 |
| ITL | 10 | 0.3 | Hex | 63.7 | [C S Chen](https://pubmed.ncbi.nlm.nih.gov/?term=Chen+CS&cauthor_id=11574255) et al.,2001 |

**ALL** Anterior longitudinal ligament**, PLL** Posterior longitudinal ligament**, LF** Ligamentum favum**, CL** Capsular ligament**; ISL** Interspinous ligament**, SSL** Supraspinal ligament**; ITL** Intertransverse ligament
