# Supplementary material for: Biomechanical assessment of unilateral/bilateral lumbar spondylolysis with and without muscle weakness using finite element analysis
Source: Heliyon. 2025 Feb 12;11(4):e42647. doi: 10.1016/j.heliyon.2025.e42647 (PMC11891713; doi:10.1016/j.heliyon.2025.e42647)
Supplement: Multimedia component 2 [file mmc2.docx]

**Supplementary table. 2** Element and node numbers for six different mesh resolutions.

| Unit size |  | Element number | Node number | von-Mises(MPa) |
| --- | --- | --- | --- | --- |
| system |  | 94289 | 49177 | 29.389 |
| 5mm |  | 127355 | 69359 | 31.769 |
| 4mm |  | 17785 | 99598 | 34.683 |
| 3mm |  | 300350 | 176279 | 31.444 |
| 2.5mm |  | 433561 | 262843 | 39.823 |
| 2mm |  | 729910 | 460514 | 30.925 |
