# Supplementary material for: Biomechanical assessment of unilateral/bilateral lumbar spondylolysis with and without muscle weakness using finite element analysis
Source: Heliyon. 2025 Feb 12;11(4):e42647. doi: 10.1016/j.heliyon.2025.e42647 (PMC11891713; doi:10.1016/j.heliyon.2025.e42647)
Supplement: Multimedia component 3 [file mmc3.docx]

**Supplementary table. 3** Material properties sensitivity analysis in RoM (degree)

|  |  | The linearized basic model | The nonlinear model | The low-value model | The high-value model |
| --- | --- | --- | --- | --- | --- |
| L1 |  | 48.439 | 50.299 | 53.031 | 44.88 |
| L2 |  | 39.823 | 41.498 | 43 | 37.499 |
| Difference |  | 8.616 | 8.801 | 10.031 | 7.381 |

Material properties sensitivity analysis in Facet joint force (N)

|  |  | The linearized basic model | The nonlinear model | The low-value model | The high-value model |
| --- | --- | --- | --- | --- | --- |
| L1 |  | 3.06 | 3.25 | 4.1 | 3.1 |
| L2 |  | 8.14 | 8.31 | 8.92 | 7.3 |
| Difference |  | 5.08 | 5.06 | 4.82 | 4.2 |
