# Supplementary material for: Biomechanical assessment of unilateral/bilateral lumbar spondylolysis with and without muscle weakness using finite element analysis
Source: Heliyon. 2025 Feb 12;11(4):e42647. doi: 10.1016/j.heliyon.2025.e42647 (PMC11891713; doi:10.1016/j.heliyon.2025.e42647)
Supplement: Multimedia component 4 [file mmc4.docx]

|  | Segment | Normal | Unilateral incomplete | Unilateral | Bilateral |
| --- | --- | --- | --- | --- | --- |
| Flexion | L4-L5 | **10.15** | **6.65** | **6.6** | **6.8** |
|  | L5-S1 | **2.88** | **6.32** | **6.3** | **6.5** |
|  |  |  |  |  |  |
| Extension | L4-L5 | **4.25** | **4.35** | **5.72** | **4.32** |
|  | L5-S1 | **4.08** | **4.6** | **3.5** | **7.82** |
|  |  |  |  |  |  |
| Left bending | L4-L5 | **4.27** | **4.26** | **4.25** | **4.15** |
|  | L5-S1 | **2.92** | **3.25** | **3.52** | **4.42** |
|  |  |  |  |  |  |
| Right bending | L4-L5 | **4.03** | **4** | **4** | **4.15** |
|  | L5-S1 | **2.78** | **2.73** | **3.05** | **4.42** |
|  |  |  |  |  |  |
| Left Torsion | L4-L5 | **1.35** | **1.37** | **1.39** | **3.46** |
|  | L5-S1 | **1.18** | **1.23** | **1.27** | **3.54** |
|  |  |  |  |  |  |
| Right Torsion | L4-L5 | **1.96** | **1.78** | **0.14** | **3.52** |
|  | L5-S1 | **1.32** | **2** | **4.07** | **3.64** |
