# Supplementary material for: Biomechanical assessment of unilateral/bilateral lumbar spondylolysis with and without muscle weakness using finite element analysis
Source: Heliyon. 2025 Feb 12;11(4):e42647. doi: 10.1016/j.heliyon.2025.e42647 (PMC11891713; doi:10.1016/j.heliyon.2025.e42647)
Supplement: Multimedia component 5 [file mmc5.docx]

|  | Segment | Normal | | Unilateral incomplete | | Unilateral | | Bilateral | |
| --- | --- | --- | --- | --- | --- | --- | --- | --- | --- |
|  | Muscle strength | 100% | 50% | 100% | 50% | 100% | 50% | 100% | 50% |
| Flexion | L4-L5 | **5.6** | **8.52** | **6** | **9.87** | **6.4** | **11.56** | **7.18** | **11.5** |
|  | L5-S1 | **12** | **9.5** | **12.5** | **11.51** | **14.96** | **11.8** | **18.47** | **11.36** |
|  |  |  |  |  |  |  |  |  |  |
| Extension | L4-L5 | **2.95** | **6.34** | **3** | **11.73** | **3.48** | **9.92** | **5.52** | **10.21** |
|  | L5-S1 | **13.68** | **12.3** | **25.66** | **15.31** | **18.52** | **8.45** | **21.45** | **20.95** |
|  |  |  |  |  |  |  |  |  |  |
| Left Bending | L4-L5 | **4.33** | **4.14** | **4.5** | **4.3** | **7.27** | **6.97** | **8.12** | **7.8** |
|  | L5-S1 | **6.47** | **5.42** | **6** | **5.62** | **8.58** | **6.95** | **9.7** | **9** |
|  |  |  |  |  |  |  |  |  |  |
| Right Bending | L4-L5 | **4.06** | **4.22** | **4.81** | **5.86** | **6.83** | **7.1** | **9.12** | **9.12** |
|  | L5-S1 | **6.78** | **4.5** | **6.07** | **5.02** | **8.31** | **7.4** | **9.16** | **8.98** |
|  |  |  |  |  |  |  |  |  |  |
| Left Torsion | L4-L5 | **2.15** | **4.88** | **2.41** | **2.75** | **8.97** | **7.82** | **2.2** | **6.287** |
|  | L5-S1 | **4.13** | **3.79** | **6** | **2** | **8.1** | **7.24** | **14.84** | **12.62** |
|  |  |  |  |  |  |  |  |  |  |
| Right Torsion | L4-L5 | **3.28** | **3** | **4** | **3.38** | **3.9** | **5.46** | **4.5** | **6.18** |
|  | L5-S1 | **2.97** | **2.38** | **3.41** | **2.92** | **2.72** | **2.22** | **8.32** | **7.11** |
